# Aging research: A field grows up

**DOI:** 10.1371/journal.pbio.3002132

**Published:** 2023-05-30

**Authors:** Coleen T. Murphy

**Affiliations:** 1 Lewis-Sigler Institute for Integrative Genomics, Princeton University, Princeton, New Jersey, United States of America; 2 Department of Molecular Biology, Princeton University, Princeton, New Jersey, United States of America

## Abstract

Breakthroughs in the longevity field over the past few decades have led to major shifts in how we attack the problem of aging. This Perspective tackles the most important of these shifts in our perspectives, aims, and approaches that will likely guide future research.

## Teaser

Breakthroughs in the longevity field over the past few decades have led to major shifts in how we attack the problem of aging. What have been the most important of these shifts in our perspectives, aims, and approaches that will likely guide future research?

This article is part of the *PLOS Biology* 20th Anniversary Collection.

When I joined the longevity field, there had already been a shift from simply observing animals as they age to instead identifying regulators that could greatly alter lifespan, thanks to pioneering invertebrate genetic studies in the 1990s and early 2000s that discovered most well-conserved longevity pathways, particularly caloric restriction and the insulin/IGF-1 and TOR pathway signaling pathways [1,2]. The downstream transcriptional targets of these pathways were identified through transcriptional studies, and the roles of these conserved signaling genes were confirmed in mice and associated with exceptional human longevity through genome-wide studies [3].

What has changed in the past two decades? There have been at least three major shifts the aging/longevity research that will shape the field in the years to come.

The first shift is in the perspective of regulation: the concept that aging is indeed regulated, and not simply the result of accumulated damage. Once the regulators of longevity were found, there was still a general notion that these pathways primarily determine levels of cell autonomous damage repair. As molecular regulators of longevity and their networks have been identified, it has become clear that non-cell autonomous signaling coordinates rates of aging and response to damage across cells and tissues [4]. Today, we better understand that for almost every cell and organelle [5], there is a system of information integration and signaling output, in addition to the cell autonomous effects that were found previously. These signals may arise in response to cues that anticipate future needs, particularly to tune reproduction to available nutrients or to coordinate responses to stresses on an organism-wide basis [6]. Sentinel tissues, like neurons, glia, and liver, signal to the rest of the body to coordinate pathways across tissues, rather than each tissue being regulated completely independently [7]. In the future, the acknowledgment that these signals are integrated and can affect the body systemically will shape the types of therapeutics we develop, focusing on whole-body versus tissue-specific approaches, depending on the problem being solved.

Additionally, the role of reproduction in the regulation of longevity is better understood; in contrast to the concept of direct resource allocation limiting an animal’s lifespan, it is now clear that regulatory systems react to environmental changes, anticipating how the animal should adjust its energy usage in response to changes in nutrients and stresses. Rather than a direct “trade off” in resources, competing pro-longevity and anti-longevity signals ultimately determine rates of both reproduction and aging in anticipation of predicted future needs, based on sensing of external conditions [8]. As humans, we do not necessarily associate aging with reproduction; we are not having dozens of children and our reproductive cessation (at least for women, and for most men) occurs long before other age-related declines, but the two are linked by the most important longevity-regulating pathways [6,9]. In fact, it is reasonable to view most “longevity” pathways as reproduction optimization pathways; the regulation of lifespan cannot be decoded without understanding its relationship to reproduction. Understanding how and when these systems communicate will affect how we approach the development of both reproductive and aging interventions.

A second large shift is in the aims of the field, from lifespan to healthspan [10]. While maximum lifespan is still often the focus of the popular press, there is growing recognition that treating aging and age-related diseases are not mutually exclusive goals. Therefore, better understanding of how metabolic disorders, frailty, cardiovascular diseases, cognitive decline, reproductive aging, and other age-related changes are regulated might not only yield treatments for those disorders, but might ultimately increase lifespan as well. Maintaining functions with age may not only have a great impact on quality of life, but also may help us find treatments that generally slow aging. Translation from genetic model systems, including new models such as the turquoise killifish [11], will be instrumental in understanding how aging-related diseases can be slowed. I am particularly excited that normal cognitive aging—in the absence of neurodegenerative disease—is being recognized as an important aspect of healthy aging that needs more attention. Similarly, reproductive aging and women’s health are finally on the scene: several initiatives have recognized not only that reproductive cessation is the earliest aging phenotype, but also that menopause itself (which is about 15 years later than reproductive aging, so not interchangeable) can cause aging, and that mid-life health may ultimately determine future longevity. This, plus the acknowledgment that female animals are important to study in all aspects of biology, may lead to the end of ignoring 50% of the aging population’s needs.

A third shift is the translational focus of the longevity field, from an almost entirely academic endeavor to one that is being taken up by industry and clinics—that is, the findings we have made in academic labs are on the verge of becoming actual aging treatments. In the most immediate future, large-scale clinical trials of some of the best-studied longevity drugs (e.g., rapamycin and metformin) and testing of dietary interventions and mimetics may lead to aging treatments. New biotech companies have sprung up with a wide range of goals, from repurposing already-approved drugs for new aging treatments [12] and exploring how the pathways we have discovered over the past 20 years might be harnessed to treat aging, to high-throughput and AI-driven approaches to search for new candidate aging drugs. Circulating blood factors first identified in parabiosis experiments, drugs that target senescent cells, and cell reprogramming and regeneration approaches [13] have moved from concepts to testing, while molecular clocks are beginning to be used as diagnostics. The fact that treatments for age-related diseases may reveal longevity drugs is being used smartly by companies: recognizing that the FDA does not yet view aging as a disease, age-related diseases are being used as proxies for aging in drug trials; this approach may both treat those age-related diseases and speed up longevity drug development. New gene editing approaches and tools borrowed from the stem cell and immunology fields hold great promise for treating aging, age-related diseases, and segmental progerias.

Luckily, we have finally matured beyond asking whether it is right to study aging, as it is being increasingly recognized that efforts to slow aging will be broadly beneficial; in fact, some of those approaches will help those with other disorders (e.g., muscle diseases, menopause and mid-life issues, and neurodegenerative diseases). Instead, we can ask, which of the multiple approaches being tested now will have the greatest impacts on our lives in the foreseeable future, and how can we all benefit?
